# Association between Visceral Fat and Brain Structural Changes or Cognitive Function

**DOI:** 10.3390/brainsci11081036

**Published:** 2021-08-04

**Authors:** Naoki Ozato, Shinnichiro Saitou, Tohru Yamaguchi, Mitsuhiro Katashima, Mina Misawa, Songee Jung, Kenta Mori, Hiromitsu Kawada, Yoshihisa Katsuragi, Tatsuya Mikami, Shigeyuki Nakaji

**Affiliations:** 1 Department of Active Life Promotion Sciences, Graduate School of Medicine, Hirosaki University, Hirosaki City 036-8562, Japan; katashima.mitsuhiro@kao.com (M.K.); mori.kenta@kao.com (K.M.); katsuragi.yoshihisa@kao.com (Y.K.); 2 Health & Wellness Products Research Laboratories, Kao Corporation, Tokyo 131-8501, Japan; yamaguchi.tohru@kao.com (T.Y.); kawada.hiromitsu@kao.com (H.K.); 3 Biological Science Research Laboratories, Kao Corporation, Tokyo 131-8501, Japan; saito.shinichiro@kao.com; 4 COI Research Initiatives Organization, Graduate School of Medicine, Hirosaki University, Hirosaki City 036-8562, Japan; m_misawa@hirosaki-u.ac.jp (M.M.); jonsoni@hirosaki-u.ac.jp (S.J.); 5 Innovation Center for Health Promotion, Hirosaki University Graduate School of Medicine, Hirosaki City 036-8562, Japan; tmika@hirosaki-u.ac.jp; 6 Department of Social Medicine, Graduate School of Medicine, Hirosaki University, Hirosaki City 036-8562, Japan; nakaji@hirosaki-u.ac.jp

**Keywords:** visceral fat area, cognitive function, dementia, elderly, cardiovascular disease, risk

## Abstract

Visceral fat accumulation is an independent risk factor for cardiovascular disease and mortality. Visceral fat is a causal risk factor for hypertension and type 2 diabetes, which was reported as one of the risk factors for dementia. Visceral fat areas (VFA) might be clinically important to prevent dementia; however, the association between VFA and cognitive function in the elderly remains unknown. We aimed to evaluate the association between brain structural abnormalities using magnetic resonance imaging (MRI) and VFA, and the association between cognitive function and VFA, in the elderly. A total of 2364 healthy individuals were enrolled, and we excluded those diagnosed with dementia. Participants were divided into a high-VFA and a low-VFA group based on median VFA. The high-VFA group had significantly lower cognitive function than the low-VFA group (*p* = 0.025), after adjustment for related factors using a linear regression model. Regarding brain structure in MRI, VFA remained significantly associated with white matter lesions (odds ratio (OR), 1.90; 95% confidence interval (1.33–2.70); adjusted *p* < 0.001) and perivascular space (OR, 1.28; 95% confidence interval (1.02–1.61); adjusted *p* = 0.033). Further follow-up studies are needed, but reducing visceral fat might be important, not only to prevent cardiovascular disease but also to prevent dementia.

## 1. Introduction

Dementia has been considered for some time to be neither preventable nor treatable; however, it has also been reported to be theoretically preventable [1]. In preventing dementia, the importance of prevention at the mild cognitive impairment (MCI) stage is now increasing. Approximately 40% of MCI patients develop dementia within 5 years [2], while 15–40% return to a healthy state [3]. Therefore, finding the factors that influence cognitive function is imperative.

Obesity in midlife, a preventable factor [1], is associated with greater cognitive decline and increased risk of dementia [4,5]. Moreover, several pathological alterations associated with obesity, such as insulin resistance or mitochondrial dysfunction, have been related to pathological processes of dementia [6]. However, the association between late-life obesity and dementia is inconclusive [7,8,9,10,11,12]. Some studies have even identified late-life obesity as a protective state for dementia [8,11,12], hence the “obesity paradox” regarding dementia risk. This inconclusive observed association has been attributed to unintentional weight loss, which is related to other comorbidities or a sign of subclinical dementia, as well as to several methodologic issues, such as the use of body mass index (BMI), which is an imperfect measure of body composition in the elderly [13]. This suggests that the association should be studied by body composition data, not by a physical index such as BMI.

Visceral fat accumulation is an independent risk factor for cardiovascular disease [14,15] and mortality [16,17,18]. Furthermore, Mendelian randomization analysis showed visceral fat to be a causal risk factor for hypertension and type 2 diabetes [19], which was reported as one of the risk factors for dementia [1]. Thus, visceral fat areas (VFA) might be clinically important to prevent dementia. Some cross-sectional studies showed that high-VFA individuals had a significantly higher prevalence of MCI [20,21]. However, others have shown that there is no significant association between VFA and MCI [22] or cognitive function [23]. Furthermore, a recent study of more than 5000 Japanese individuals showed that visceral fat accumulation was protective against MCI [24]. Therefore, the association between visceral fat and cognitive function is inconclusive, suggesting that research using more objective indicators is necessary.

It is well known that cognitive impairment fluctuates with brain structural changes, such as atrophy [25] and white matter lesions [26]. Furthermore, various structural abnormalities have been reported to occur in the brain in patients with dementia [27]. In the present baseline study, we investigated the relationship between brain structural abnormalities using magnetic resonance imaging (MRI) and VFA, in addition to the relationship between cognitive function and VFA.

## 2. Materials and Methods

### 2.1. Design, Participants, and Ethics

The Japan Prospective Studies Collaboration for Aging and Dementia’s (JPSC-AD) Hirosaki study, one study field of the JPSC-AD [28], was launched in 2016 as a 12-year prospective cohort study, aiming to elucidate the causative factors of dementia and to develop preventative methods. Visceral fat was measured as a unique item of the JPSC-AD Hirosaki study. Therefore, one of the aims of this study was to evaluate the effect of VFA on dementia.

**Figure 1:**
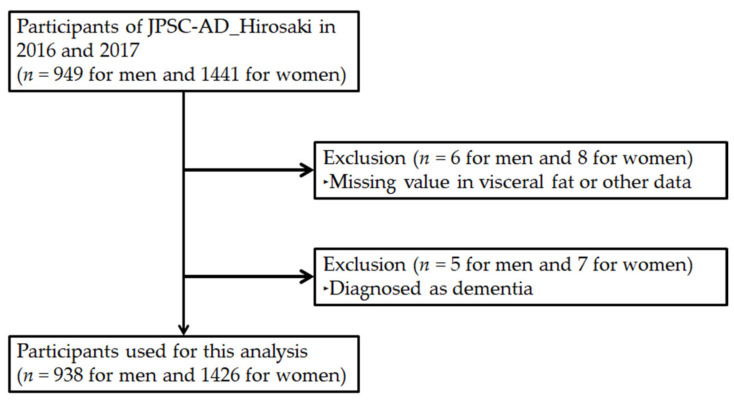
Study flow for selection of the participants. A total of 2364 participants were enrolled in the analysis. JPSC-AD, Japan Prospective Studies Collaboration for Aging and Dementia.

A baseline study was conducted in 2016 and 2017. Participants were men and women, at least 64 years of age, living in Hirosaki City, Aomori Prefecture, Japan. In this baseline study, 2390 individuals participated in the health check-up (Figure 1). Of these, 14 participants did not complete the clinical assessment and were excluded from the analyses. It is well known that unintentional weight loss occurs before dementia. Therefore, in the present study, to minimize the weight loss effect upon the association, we excluded individuals who were already diagnosed with dementia (*n* = 12). Thus, 2364 individuals (937 men, 1427 women; mean age ± standard deviation, 70.3 ± 4.4 years for men, 69.8 ± 4.1 years for women) were enrolled in the present study.

The study was approved by the Ethics Committee of Hirosaki University School of Medicine and conducted in accordance with the principles of the Declaration of Helsinki (2019-064). Written informed consent was obtained from all participants prior to the study.

### 2.2. Measurements of Cognitive Function and Brain MRI Examination

Mini-mental state examination (MMSE) [29] was conducted by a clinician who is trained and experienced in its use, and was used to evaluate cognitive function. A score < 24 is frequently implemented as the abnormal cutoff value and is indicative of cognitive impairment [30]; therefore, in this study, an MMSE score < 24 was defined as cognitive impairment. The equipment for the brain MRI was set with T1WI parameters, according to the protocol of the brain MRI for the Alzheimer’s Disease Neuroimaging Initiative (ADNI) study [31], at all research sites. In addition, brain MRI data were standardized using the MRI Phantom, Human Phantom, and ADNI Phantom to correct geometric distortions among the different equipment. Diagnoses of atrophy, leukoaraiosis, periventricular hemorrhage (PVH) grade, perivascular, and hemorrhage were outsourced to the Center for Advanced Functional Imaging in Medicine (Aomori, Japan) according to the instructions of the vendors. The diagnosis criteria of each brain disorder are described in Table 1.

### 2.3. Diagnosis of Depression, Hypertension, Hyperlipidemia, and Diabetes

The Geriatric Depression Scale (GDS) short version was used to assess depressive symptoms. Depressive symptoms were defined as a GDS score of ≥ 6 or the current use of antidepressant medication. The subjects with depressive symptoms underwent a second screening survey of depression by using the Mini-International Neuropsychiatric Interview, where depression was diagnosed according to the criteria of the Diagnostic and Statistical Manual of Mental Disorders—fourth edition (DSM—IV). Hypertension was defined as blood pressure ≥ 140/90 mmHg, or the use of antihypertensive drugs [34]. Hyperlipidemia was defined as low-density lipoprotein (LDL) cholesterol ≥ 140 mg/dL, high-density lipoprotein (HDL) cholesterol < 40 mg/dL, triglycerides ≥ 150 mg/dL, or the use of antihyperlipidemic drugs [35]. Diabetes was defined as fasting serum glucose ≥ 126 mg/dL, HbA1c ≥ 6.5%, or the use of antidiabetic drugs [36].

### 2.4. Measurements of Other Items

All participants fasted for at least 9 h prior to undergoing the measurements. The visceral fat meter EW-FA90 (Panasonic Corporation, Osaka, Japan), authorized as a medical device in Japan (No. 22500BZX00522000), was used to measure VFA. The results of this device highly correlate with the value obtained using computed tomography [37], which is authorized as the gold standard for VFA measurement. The following clinical characteristics were also measured: body weight; height; BMI; waist circumference; diastolic blood pressure; systolic blood pressure; glycated hemoglobin; fasting serum glucose; total serum cholesterol concentration; triglycerides; LDL cholesterol; and HDL cholesterol. All laboratory tests were outsourced to LSI Medience Co. (Tokyo, Japan), according to our instructions. Blood samples were collected in the morning from the peripheral veins of the participants. Smoking habits and sleep time (hours/d) were determined from questionnaires.

### 2.5. Statistical Analysis

The participants’ characteristics are presented as mean ± standard deviation (SD). Groups (high-VFA group and low-VFA group) were compared using the Wilcoxon rank-sum test. The association between VFA and cognitive function using MMSE was assessed by Spearman’s correlation coefficient. The association between VFA status and cognitive function was adjusted using a linear regression model, with an MMSE score as an objective variable, and VFA and covariates including age, sex, and lifestyle habits as explanatory variables. Fisher’s exact test was used to determine whether there were significant differences in the incidence of cognitive impairment between the high- or low-VFA groups. Furthermore, Fisher’s exact test was used to determine whether there were significant differences in the incidence of each brain disorder between the high- or low-VFA groups. To exclude the effects of covariates such as age and lifestyle habits, we calculated odds ratios (ORs) and 95% confidence intervals (CIs) of each brain disorder by VFA status using a logistic regression model.

Statistical tests were two-tailed. Values of *p* < 0.05 were considered statistically significant. We used R statistical software and environment (version 4.0.5).

## 3. Results

### 3.1. Baseline Characteristics of the Study Cohort

Study participant characteristics (N = 2364, 60% women) are shown in Table 2. The proportion of overweight individuals defined as BMI ≥ 25 was 30.2% for men and 18.8% for women. The obesity rate, defined as BMI ≥ 30, was 4.0% for men and 3.0% for women. The proportions are comparable to the 2010 Japanese national survey (overweight and obesity rate in subjects 30–69 years of age: 33.5% for men and 20.5% for women) [38]. Mean VFA was 86.0 ± 41.2 cm^2^, lower than the value defined by the Japan Society for the Study of Obesity [39] as visceral obesity (≥100 cm^2^). The frequencies of participants smoking or drinking alcohol were 7.0% and 46.7%, respectively.

The participants were divided into two groups based on the median VFA: a high-VFA (260 men and 961 women) and low-VFA group (465 men and 678 women; see Table 2). Concerning the metabolic risk factors, the Wilcoxon rank-sum test showed that glucose (*p* < 0.001), glycated hemoglobin (HbA1c, *p* = 0.001), diastolic blood pressure (DBP, *p* < 0.001), systolic blood pressure (SBP, *p* < 0.001), triglyceride (TG) (*p* < 0.001), and LDL cholesterol (*p* < 0.001) levels were significantly higher in the high-VFA group than in the low-VFA group, while the HDL cholesterol levels were significantly lower in the high-VFA group (*p* < 0.001). Therefore, the prevalence of hypertension, hyperlipidemia, or diabetes patients was significantly higher in the high-VFA group than in the low-VFA group (all *p* < 0.001). Concerning dietary habits, energy and alcohol intake were significantly higher in the high-VFA group than in the low-VFA group (*p* = 0.002 and *p* < 0.001, respectively). The high-VFA group had a higher energy intake and the prevalence of drinking alcohol was significantly higher (both *p* < 0.001). Furthermore, concerning smoking habits, the prevalence of smoking was significantly higher in the high-VFA group than in the low-VFA group (*p* = 0.011).

### 3.2. Association between Cognitive Function and Visceral Fat

**Figure 2:**
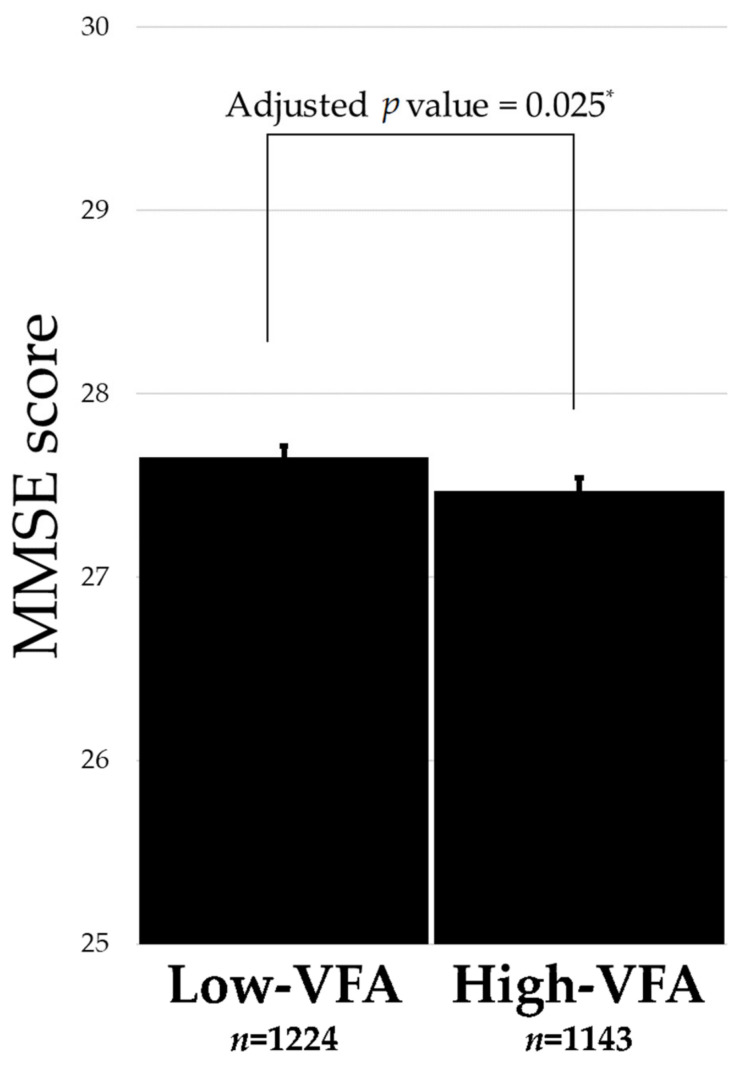
Association between cognitive function and VFA using the mini-mental state examination (MMSE). The *p* value was calculated by analysis of variance for a linear regression model with the MMSE score as an objective variable, and covariates such as VFA, age, sex, muscle mass, education, smoking, body mass index, exercise habits, alcohol consumption, and prevalent confounding disease (depression, hypertension, hyperlipidemia, and diabetes). * *p* < 0.05

VFA was negatively and significantly associated with an MMSE score (r = −0.401 and *p* value = 0.0499; Spearman). The association between each VFA group and MMSE score is shown in Figure 2. Therefore, the *p* value was calculated by analysis of variance for a linear regression model with an MMSE score as an objective variable, and VFA status and covariates such as age, sex, muscle mass, education, smoking, BMI, exercise habits, alcohol consumption, and prevalent confounding disease (depression, hypertension, hyperlipidemia, and diabetes). After adjusting for related factors, the high-VFA group had significantly lower cognitive function than the low-VFA group (27.7 ± 0.07 for low-VFA group and 27.5 ± 0.07 for high-VFA group; adjusted *p* value = 0.025). Furthermore, to evaluate the association between cognitive impairment and visceral fat, the incidence of cognitive impairment according to VFA was assessed (Supplementary Table S1). The high-VFA group had a significantly higher incidence of cognitive impairment than the low-VFA group (7.3% for high-VFA group and 5.0% for low-VFA group; *p* value = 0.036).

### 3.3. Association between Brain Disorder and Visceral Fat

To evaluate the association between brain structural changes and visceral fat, the incidence of each brain disorder according to VFA was assessed (Table 3). The high-VFA group had a significantly higher incidence of brain atrophy than the low-VFA group (29.2% for high-VFA group and 19.2% for low-VFA group; *p* value < 0.001). The high-VFA group had a significantly higher incidence of white matter lesions than the low-VFA group (10.7% for high-VFA group and 6.3% for low-VFA group; *p* value < 0.001). The high-VFA group had a significantly higher incidence of PVH grade than the low-VFA group (17.1% for high-VFA group and 13.1% for low-VFA group; *p* value = 0.008). The high-VFA group had a significantly higher incidence of perivascular characteristics than the low-VFA group (27.0% for high-VFA group and 22.1% for low-VFA group; adjusted *p* value = 0.008). Furthermore, the high-VFA group had a significantly higher incidence of hemorrhage than the low-VFA group (15.0% for the high-VFA group and 11.5% for the low-VFA group; *p* value = 0.016).

**Figure 3:**
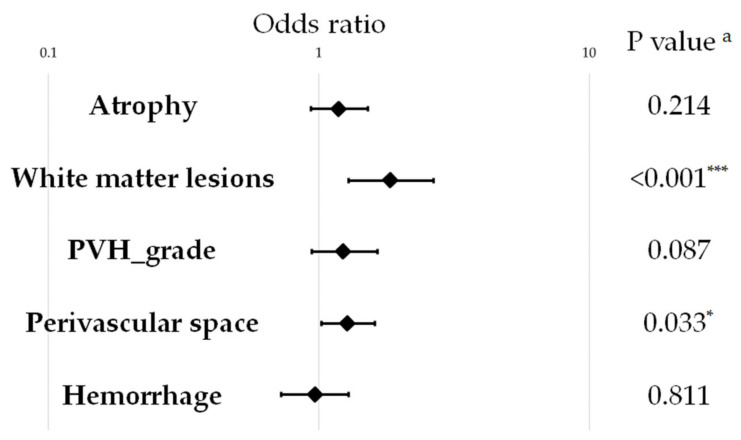
Association between brain structure and visceral fat area using magnetic resonance imaging. Data are presented as the odds ratio and 95% confidence intervals. ^a^ *p* value was adjusted using logistic regression, adjusting for age, sex, muscle mass, education, smoking, body mass index, exercise habits, alcohol consumption, and prevalent confounding diseases (depression, hypertension, hyperlipidemia, and diabetes). * *p* < 0.05; *** *p* < 0.001.

A variety of environmental factors, including age and sex, might affect the association between brain disorders and visceral fat. The results might be affected by these confounding variables. Therefore, we calculated ORs and 95% CI of each brain disorder by VFA status using logistic regression after adjusting for age, sex, muscle mass, education, smoking, BMI, exercise habits, alcohol consumption, and prevalent confounding diseases (depression, hypertension, hyperlipidemia, and diabetes) (Figure 3). Even after adjusting for confounding factors, VFA was significantly associated with white matter lesions and perivascular space (OR, 1.90; 95% CI, 1.33, 2.70; *p* value < 0.001 for white matter lesions and OR, 1.28; 95% CI, 1.02, 1.61; *p* value = 0.033 for perivascular space). However, there were no significant associations between VFA and atrophy, PVH grade, or hemorrhage after adjusting for confounding variables (*p* = 0.214, *p* = 0.087, and *p* = 0.811, respectively). To assess regression, we calculated ORs and 95% CI of each brain disorder by VFA value using logistic regression after adjusting for age, sex, muscle mass, education, smoking, BMI, exercise habits, alcohol consumption, and prevalent confounding disease (depression, hypertension, hyperlipidemia, and diabetes) (Supplementary Table S2). Similar results were observed for white matter lesions and perivascular space. However, VFA was significantly associated with PVH grade, suggesting that further studies are needed.

## 4. Discussion

This study reports an association between VFA and cognitive function or brain disorders in a relatively large number of elderly participants. The association between VFA and cognitive function was inconclusive in previous studies. In this study, we found that high-VFA participants suffered significantly lower cognitive function after adjusting for related factors (Figure 2), and similar results have been confirmed in prior studies using MMSE [20] or the Test-Your-Memory cognitive screening tool [21] to assess cognitive functioning. However, contrasting results have also been reported using the National Center for Geriatrics and Gerontology-Functional Assessment Tool [24]. One reason for the inconsistent views may be that the methods of evaluating cognitive function differ from study to study. Furthermore, the mean VFA of this study was 86.0 ± 41.2 cm^2^, whereas, in a prior study [20], the mean VFA was 121.7 ± 46.3 cm^2^ for under 70-year-olds and 139.8 ± 63.7 cm^2^ for over 70-year-olds. In another study [21], VFA level was used, and mean VFA was not reported. In contrast, according to Chiba et al. [24], cognitive decline was suppressed in women with high visceral fat. The mean VFA for women was 63.3 cm^2^ and the interquartile range was 44.0–85.3 cm^2^, suggesting that almost all women were under 100 cm^2^, which is defined as the visceral obesity cutoff by the Japan Society for the Study of Obesity [39]. Furthermore, in studies that included participants with a mean VFA of 202.8 ± 86.0 cm^2^ for men and 150.2 ± 67.1 cm^2^ for women [22], no significant relationship between VFA and cognitive decline was confirmed. To summarize these results, in the subject group with visceral fat mass of approximately 100 cm^2^, the visceral fat mass was inversely correlated with cognitive function, as observed in this study [20], and in the subject group with visceral fat mass of less than 100 cm^2^, the visceral fat mass was positively correlated with cognitive function [24]. In the subject group with visceral fat mass over 100 cm^2^, the visceral fat mass was not correlated with cognitive function [22]. These results suggest that visceral fat mass of approximately 100 cm^2^ might be a cutoff point in the association between VFA and cognitive function in the elderly, although further research is needed.

Cognitive impairment fluctuates with brain structural changes such as atrophy [25] and white matter lesions [26]. Therefore, we assessed the association between VFA and brain disorders, and found that VFA was significantly associated with the incidence of white matter lesions and perivascular space, even after adjusting for confounding factors (Figure 3). The incidence of white matter lesions is reported to be significantly and positively associated with high VFA in type 2 diabetes patients [40]. However, it is not significantly associated with high VFA in healthy, middle-aged participants [41], suggesting that the association in middle age is conclusive. In our study, the incidence of white matter lesions was significantly associated with high VFA in the elderly. As for the perivascular space, a similar result has been obtained in middle-aged patients [41]. These results suggest that reducing visceral fat from middle age might be important for brain health, or that reducing for visceral fat in old age might be important for brain health, although further investigation is needed. All previous studies [40,42], as well as this present study, were cross-sectional, and we look forward to the results of our prospective cohort study. In our study, the incidence of atrophy, PVH grade, and hemorrhage was significantly higher in the high-VFA group (Table 3). However, after adjusting for related factors, this was not significant. These results suggest that sex, age [43], metabolic syndrome factors [43], and muscle mass [44] may have influenced the association. In fact, cerebral atrophy is reported in diabetes patients [45]. Significant associations have been reported between visceral fat and incidence of atrophy [46], perivascular characteristics [41], and hemorrhage [47]. However, in our study, after confounding by related factors, there was no significant association.

In the present study, we accurately studied the relationship between cognitive function and visceral fat, and patients diagnosed with dementia were excluded. Statistical adjustments were made for confounding factors, such as age, BMI, metabolic factors, smoking habits, and alcohol intake. We found that VFA was a factor independently associated with cognitive function and brain disorders. Furthermore, VFA was measured using a visceral fat meter, EW-FA90, which is an authorized medical device in Japan.

A limitation of the present study is that we could not assess whether high VFA is a risk factor for future cognitive decline or brain structural changes, because this was a cross-sectional and not a longitudinal cohort study. Furthermore, recent studies have demonstrated that poor cognitive control influences obesity-related behaviors [48], and there are many drugs that affect cognitive function; however, this was not investigated in the present study.

## 5. Conclusions

Visceral fat was negatively and significantly associated with cognitive function in elderly Japanese patients. Furthermore, visceral fat was negatively and significantly associated with white matter lesions and perivascular space, suggesting that reducing visceral fat might be important, not only for cardiovascular disease, but also for brain health.

## Figures and Tables

**Table 1:** Diagnosis criteria for brain disorders using MRI.

Characteristics	Criteria
Atrophy	“Boundary” or “yes”
White matter lesions [32,33]	Beginning confluence of foci (≥3 mm)
PVH grade [32,33]	Smooth “halo”
Perivascular	Judge by basal ganglia level 1 slice (≥6)
Hemorrhage	“Yes”

MRI, magnetic resonance imaging; PVH, periventricular hemorrhage.

**Table 2:** Characteristics of the study participants according to VFA.

Characteristics	All	Low-VFA	High-VFA	*p* Value ^a^
Number (men/women)	2364 (938/1426)	1221 (260/961)	1143 (465/678)	
Age (y)	70.0 ± 4.2	70.0 ± 4.2	70.1 ± 4.2	0.573
Height (cm)	157.8 ± 8.4	155.2 ± 7.2	160.7 ± 8.6	<0.001 ***
Metabolic risk factors				
BMI (kg/m^2^)	22.8 ± 3.0	21.0 ± 2.1	24.7 ± 2.7	<0.001 ***
Waist circumference (cm)	77.8 ± 9.4	71.2 ± 6.0	84.8 ± 7.1	<0.001 ***
VFA (cm^2^)	86.0 ± 41.2	54.7 ± 18.0	119.5 ± 31.5	<0.001 ***
Glucose (mmol/L)	5.2 ± 1.0	5.0 ± 0.8	5.4 ± 1.1	<0.001 ***
HbA1c (%)	5.8 ± 0.6	5.7 ± 0.5	5.9 ± 0.7	<0.001 ***
DBP (mmHg)	83.2 ± 12.1	82.2 ± 12.1	84.2 ± 12.0	<0.001 ***
SBP (mmHg)	150.5 ± 21.9	148.4 ± 22.8	152.7 ± 20.5	<0.001 ***
TG (mmol/L)	1.1 ± 0.6	1.0 ± 0.5	1.3 ± 0.7	<0.001 ***
HDL cholesterol (mmol/L)	1.8 ± 0.4	1.9 ± 0.4	1.6 ± 0.4	<0.001 ***
LDL cholesterol (mmol/L)	3.3 ± 0.8	3.4 ± 0.8	3.2 ± 0.8	<0.001 ***
Lifestyle habit				
Energy intake (kcal)	1765 ± 413	1726 ± 367	1805 ± 453	<0.001 ***
Smoking habit (yes, %)	7.0	5.6	8.5	0.011 *
Sleep time (h/d)	7.0 ± 1.2	7.1 ± 1.2	7.0 ± 1.2	0.439
Daily exercise (yes, %)	68.8	70.3	67.2	0.117
Alcohol intake (yes, %)	46.7	37.5	56.5	<0.001 ***
Disease				
Depression (%)	1.6	1.7	1.6	0.914
Hypertension (%)	79.6	72.2	87.1	<0.001 ***
Hyperlipidemia (%)	49.6	43.0	56.6	<0.001 ***
Diabetes (%)	17.0	11.7	22.7	<0.001 ***

VFA, visceral far area; BMI, body mass index; HbA1c, hemoglobin A1c; SBP, systolic blood pressure; DBP, diastolic blood pressure; TG, triglycerides; HDL, high-density lipoprotein; LDL, low-density lipoprotein. * *p* < 0.05; *** *p* < 0.001. Data represent mean ± SD. ^a^ *p* value was evaluated between low-VFA and high-VFA groups.

**Table 3:** Incidence of each brain disorder according to VFA.

Characteristics	Low-VFA (*n* = 1224)	High-VFA (*n* = 1143)	*p* Value ^a^
Atrophy	19.2%	29.2%	<0.001 ***
Non-atrophy	80.8%	70.8%	
White matter lesions	6.3%	10.7%	<0.001 ***
Non-white matter lesions	93.7%	89.3%	
PVH grade	13.1%	17.1%	0.008 **
Non-PVH grade	86.9%	82.9%	
Perivascular	22.1%	27.0%	0.008 **
Non-perivascular	77.9%	73.0%	
Hemorrhage	11.5%	15.0%	0.016 *
Non-hemorrhage	88.5%	85.0%	

VFA, visceral far area; PVH, periventricular hemorrhage. * *p* < 0.05; ** *p* < 0.01; *** *p* < 0.001. ^a^ Fisher’s exact test was used between high- or low-VFA groups.

## Data Availability

All data generated during and analyzed during the current study are included in this article and its supplementary information files or are available from the corresponding author upon reasonable request.

## Supplementary Materials

The following are available online at https://www.mdpi.com/article/10.3390/brainsci11081036/s1, Table S1: Association between cognitive impairment and visceral fat area; Table S2: Association between brain structure and visceral fat area.
