# Supplementary material for: Association between Visceral Fat and Brain Structural Changes or Cognitive Function
Source: Brain Sci. 2021 Aug 4;11(8):1036. doi: 10.3390/brainsci11081036 (PMC8391376; doi:10.3390/brainsci11081036)
Supplement: Supplementary file 1 [file brainsci-11-01036-s001.zip › brainsci-1320517-supplementary.pdf]

Table S1. Association between cognitive impairment and visceral fat area.

Characteristic	Low-VFA, % (N=1224)	High-VFA, % (N = 1143)	P value [a]
MMSE≥24	95.0	92.7	0.036*
MMSE<24	5.0	7.3	

MMSE, mini-mental state examination; VFA, visceral fat area.

Table S2. Association between brain structure and visceral fat area.

	Odds ratio	95% CI	P Value [a]
Atrophy	1.002	0.998–1.007	0.320
White matter lesions	1.006	1.001–1.011	0.011 *
PVH_grade	1.004	1.001–1.008	0.013 *
Perivascular space	1.004	1.001–1.008	0.004 **
Hemorrhage	0.997	0.994–1.001	0.181

[a] P value was derived using a logistic regression model adjusting for age, sex, muscle mass, education, smoking, body mass index, exercise habits, alcohol consumption, and prevalent confounding diseases (depression, hypertension, hyperlipidemia, and diabetes).

PVH, periventricular hemorrhage; CI, confidence interval.

* P < 0.05; ** P < 0.01; *** P < 0.001.
